# Cost-effectiveness of cognitive therapy as an early intervention for post-traumatic stress disorder in children and adolescents: a trial based evaluation and model

**DOI:** 10.1111/jcpp.12851

**Published:** 2017-12-02

**Authors:** James Shearer, Nestor Papanikolaou, Richard Meiser-Stedman, Anna McKinnon, Tim Dalgleish, Patrick Smith, Clare Dixon, Sarah Byford

**Affiliations:** 1 King’s Health Economics, King’s College London, London; 2 Medical Research Council Cognition and Brain Sciences Unit, University of Cambridge, Cambridge; 3 Department of Clinical Psychology, University of East Anglia, Norwich; 4 Cambridgeshire and Peterborough NHS Foundation Trust, Cambridge; 5 Institute of Psychiatry, Psychology and Neuroscience, King’s College London, London, UK

**Keywords:** Economic evaluation, post-traumatic stress disorder, cognitive therapy

## Abstract

**Background:**

Untreated post-traumatic stress disorder (PTSD) in children and adolescents is associated with a considerable economic burden on the health system, families and society. Recent research has demonstrated the potential efficacy of cognitive therapy as an early intervention for PTSD in children and adolescents. Children who experienced a single traumatic event in the previous two to six months and were randomized to cognitive therapy for PTSD (CT-PTSD) were significantly more likely to be PTSD-free compared to those randomized to usual care represented by waitlist control. The current study evaluated the economic impact of improvements in the treatment of PTSD in children and adolescents.

**Methods:**

A cost-effectiveness analysis was conducted from the national health service/personal social services perspective with outcomes expressed as quality-adjusted life years (QALYs). Patient level costs and outcomes were collected during the 11 week clinical trial and extrapolated to a three year time horizon using economic modelling methods. Uncertainty was estimated using probabilistic sensitivity analysis and assumptions were tested using one way sensitivity analysis.

**Results:**

The incremental cost-effectiveness ratio at 3 years was £2,205 per QALY with a 60%–69% probability of CT-PTSD being cost-effective compared to usual care at the UK £20,000 to £30,000 per QALY decision threshold.

**Conclusions:**

This study provides preliminary evidence for the cost-effectiveness of cognitive therapy in this treatment population. Larger pragmatic trials with longer follow-up are indicated.

## Introduction

In peace time, more than half of children and adolescents will experience, or witness, traumatic events such as violence, abuse, vehicle accidents, house fires, deaths and injuries (Copeland, Keeler, Angold, & Costello, 2007). A meta-analysis conducted in 2014 estimated that 16% of children and adolescents exposed to trauma will go on to develop post-traumatic stress disorder (PTSD) (Alisic et al., 2014). Untreated, PTSD in children and adolescents tends to have a chronic course and high comorbidity with other mental health disorders such as anxiety, depression and severe behavioural problems (Bolton, O’Ryan, Udwin, Boyle, & Yule, 2000; Fletcher, 1996). The potential economic burden of untreated childhood and adolescent PTSD includes higher lifetime health care costs, impaired quality of life for patients and their families, educational difficulties and potentially poorer employment outcomes (Makley & Falcone, 2010).

There is no established best practice for early intervention for children and adolescents at risk of PTSD after a single traumatic event (Marsac, Donlon, & Berkowitz, 2014), with concerns in the adult literature that early intervention in the first four weeks (in particular single-session psychological debriefing) may be ineffectual or even impede natural recovery (Rose, Bisson, Churchill, & Wessely, 2002). The “Acute Stress Program for Children and Teenagers” or ASPECTs study was the first study of early cognitive treatment for PTSD (CT-PTSD) in children and adolescents adapted from a successful early cognitive intervention in adults (Ehlers et al., 2003; Meiser-Stedman et al., 2017). The intervention was targeted towards children and adolescents diagnosed with PTSD, according to a developmentally sensitive algorithm, two to six months after a single traumatic event.

Information about the cost-effectiveness of effective interventions is an important part of changing clinical practice and translating research into patient benefit (Mihalopoulos et al., 2015). The objective is to compare alternative treatment options in terms of their relative costs and health gains, commonly using cost-utility analysis where cost-effectiveness is expressed as the cost per quality-adjusted life year (QALY), a generic measure of health gain combing survival time with a quality of life weighting. Cost-effectiveness can be evaluated using patient-level cost and outcome data collected during a clinical trial (Petrou & Gray, 2011a) or modelled using costs, outcomes and disease progression data from diverse sources (including trial data; Petrou & Gray, 2011b) or assessed through a combined approach where trial data are extrapolated beyond the trial time horizon to capture the longer term impact of treatment on disease progression, costs and benefits (Hughes et al., 2016). The current study takes the trial-based extrapolation approach.

We systematically reviewed the cost effectiveness literature in childhood PTSD and identified two published economic evaluations which modelled the cost-effectiveness of trauma-focussed cognitive behavioural therapy (TF-CBT) for childhood and adolescent PTSD (Gospodarevskaya & Segal, 2012; Mihalopoulos et al., 2015). TF-CBT and CT-PTSD are both CBT based therapies for the treatment of child and adolescent PTSD. TF-CBT is mainly used in chronic trauma such as sexual abuse. Gospodarevskaya and Segal (Gospodarevskaya & Segal, 2012) compared TF-CBT with antidepressants, nondirective counselling and no treatment in terms of QALYs and costs to the Australian mental health care system, for sexually abused children and adolescents with PTSD and/or depression over a 30-year time horizon. TF-CBT dominated nondirective counselling (achieving more QALYs at lower cost) and was cost-effective compared to no treatment, with costs per QALY under A$2,000, well below the Australian cost-effectiveness threshold (the amount a health provider is broadly prepared to pay for one QALY) of AU$50,000. Mihalopoulos and colleagues (Mihalopoulos et al., 2015) also modelled TF-CBT in children but over a shorter time horizon of 5 years. They estimated the cost per QALY at AU$8,900; again, well under the Australian cost-effectiveness threshold of AU$50,000.

Although both analyses concluded that TF-CBT was cost-effective from the Australian health care system perspective, these findings may not be generalizable to other health care systems or to the treatment of children and adolescents at risk of PTSD after a single acute traumatic episode. Importantly, one model concerned sexually abused young people who are at greater risk of depression and suicide later in life. Finally, both evaluations were economic models that synthesized cost, outcome and remission rates taken from different sources and applied them to a hypothetical cohort of patients. The present study is the first to estimate cost-effectiveness based on patient-level cost and effect data observed in a randomized clinical trial of CT-PTSD for children and adolescents with recent exposure to single-incident traumatic stressor (e.g. motor vehicle collision, assault).

## Methods

### Clinical trial

The ASPECTs study was an 11-week randomized waitlist controlled trial of early PTSD treatment in trauma exposed children and adolescents delivered 2–6 months after a single trauma event (Meiser-Stedman et al., 2017). The study recruited participants from Emergency Departments, community mental health teams, primary care, schools and other health clinics across the East of England region. Children and adolescents were included if they were aged 8–17 years, and met age-appropriate diagnostic criteria for PTSD; all participants met ICD-10 criteria for PTSD. Twenty nine children were recruited and randomized equally to CT-PTSD (*n* = 14) or an 11 week waitlist control group (*n* = 15). The treatment group were offered individual weekly sessions of CT-PTSD over 10 weeks delivered by trained clinical psychologists. The waitlist control group reflected usual care offered by the English National Health Service (NHS) where early intervention is not usually offered to treat children at risk of trauma related PTSD. All waitlist control patients were offered CT-PTSD at 11 weeks.

### Economic evaluation

The economic evaluation was a cost-utility analysis taking the UK National Health Service/Personal Social Services perspective for costs and using QALYs as the primary economic outcome. Resource use was collected using the Child and Adolescent Service Use Schedule (CA-SUS), designed and successfully implemented in various evaluations of child and adolescent mental health services (Goodyer et al., 2017), and clinical records for intervention contact time. Resource use associated with the index trauma such as accident and emergency department attendance was excluded. All services used were costed using nationally applicable unit costs at 2014 prices (Curtis, 2014). The intervention cost was based on the contact time multiplied by the hourly unit cost for a clinical psychologist (UK NHS Agenda for Change Band 8a) of £138 per hour including employer costs (national insurance and super-annuation), overhead costs and noncontact time (Curtis, 2014). The 14 young people in the treatment group received an average of 636.25 min of contact time (range 195–755 min) and attended an average of 8.3 sessions (range 4–10) at a mean cost of £1463.

Utility weights used to calculate QALYs were derived from the parent-completed Strengths and Difficulties Questionnaire (SDQ), a commonly used measure of mental health in children and adolescents (Goodman, 2007). SDQ scores were mapped to the Child Health Utility index 9D (CHU-9D) using a published mapping algorithm developed in a sample of 200 caregivers of young people in Australia attending child and adolescent mental health services (Furber, Segal, Leach, & Cocks, 2014). The CHU-9D is a generic measure of children’s health state preferences consisting of nine dimensions (sad, worried, pain, annoyed, tired, homework or schoolwork, daily routine, activities and sleep) rated using five levels (Stevens, 2012). The CHU-9D is recognized as a valid and responsive utility measure designed exclusively for use in children (Canaway & Frew, 2013). The algorithm mapping the SDQ to the CHU-9D performed well in predicting mean group observed utility values. Equation 1 transforms the five SDQ subscale scores into a utility value or weight which is used to calculate QALYs.

(1) $$\begin{array}{l} \text{Utility}=0.88+(-0.019\times \text{emotion})\\ \;+(-0.009\times \text{conduct})+(-0.001\times \text{hyper})\\ \;+(-0.008\times \text{peer})+(0.005\times \text{prosocial}) \end{array}$$

A QALY is calculated by multiplying survival time by a utility weight. For example, if a child lives two years with quality-of-life weighted at 0.5, the two life years are multiplied by 0.5 to yield one QALY.

### Economic model

**Figure 1:**
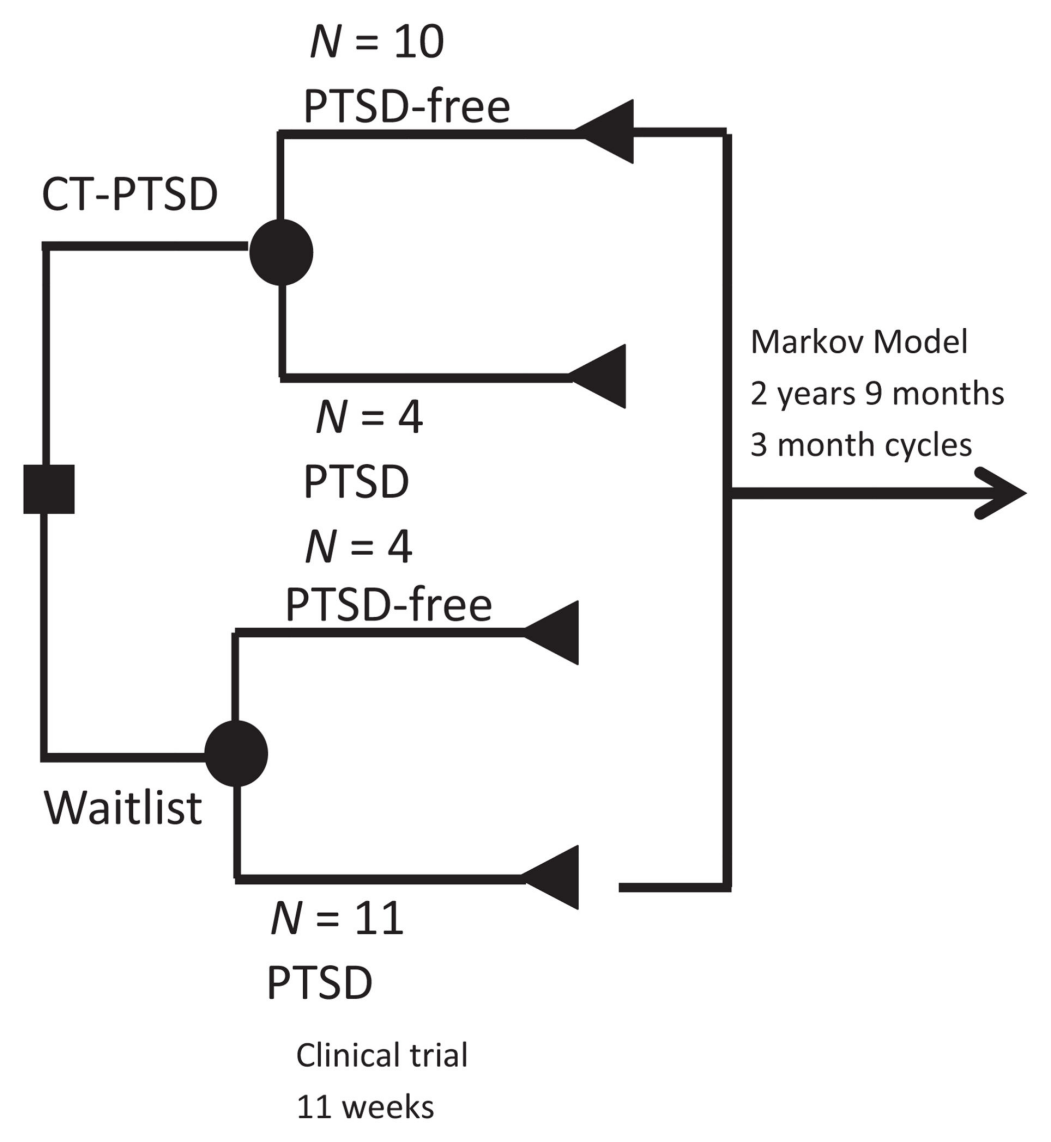
Decision model

**Figure 2:**
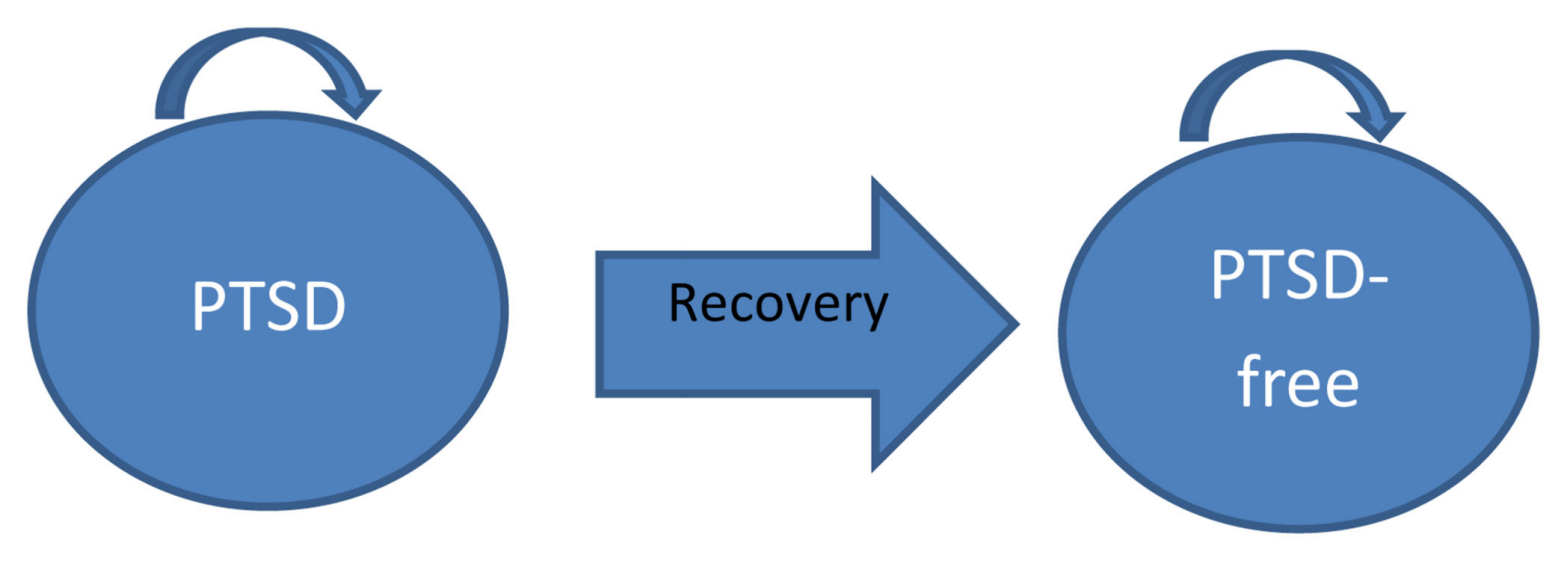
Patient flow for Markov model [Colour figure can be viewed at wileyonlinelibrary.com]

An economic model was developed to extrapolate costs and consequences expected to occur after the initial trial period. This was a Markov model with two health states defined by PTSD diagnosis (PTSD or PTSD-free). The children entered the model according to their group allocation and PTSD status at the end of the 11 week trial (see Figure 1). A time horizon of three years was selected since most natural recovery occurs within three years of an acute traumatic event (Breslau, 2009). The children move through the Markov model in three-month cycles accruing costs and QALYs depending on whether they are in the PTSD health state or the PTSD-free health state (Figure 2). The three month cycle length was an approximation of the 11 week trial period. The PTSD health state value was based on the mean costs and QALYs of children at baseline (*n* = 29). The PTSD-free health state value was based on the costs (excluding the cost of CT-PTSD) and QALYs for all children who were PTSD-free at trial follow up irrespective of group allocation (*n* = 14). Natural recovery was simulated using transition probabilities estimated from a meta-analytic study of child post-traumatic stress disorder (Hiller et al., 2016). These reflect the probability that a PTSD patient will recover at the end of each three month time cycle in the first year. Hiller et al. (2016) found prevalence of PTSD reduced by 34% between 3 and 12 months after diagnosis. The nine month probability of .34 from (Hiller et al., 2016) was converted to an instantaneous nine-month rate, divided by three to derive the three-month rate, and then the three-month rate was converted to a three-month probability of .129 as recommended by (Miller & Homan, 1994). Natural recovery was not modelled in years 2 and 3 as there was little evidence of further significant spontaneous recovery after one year (Gospodarevskaya & Segal, 2012; Hiller et al., 2016). Once recovered from a single acute traumatic event, the risk of relapse is considered very low (Hong et al., 2014) and is not considered in the model. Costs and QALYs after the first year were discounted at the UK Treasury rate of 3.5% to reflect time preferences (Shearer & Byford, 2015). The model summed total costs and QALYs for each group over 2 years and 9 months which, added to the trial based costs and QALY data, provided the data for the cost-utility analysis.

We recommend that readers unfamiliar with health economic methods refer to the following helpful primer papers designed for clinicians on economic evaluation (Shearer & Byford, 2015) and economic modelling (Petrou & Gray, 2011b).

### Analysis

The cost effectiveness of CT-PTSD relative to usual care is presented as an incremental cost-effectiveness ratio (ICER) which is the difference in mean costs divided by the difference in mean QALYs, expressed as the cost per QALY. CT-PTSD is considered cost effective from the perspective of the UK National Institute for Health and Care Excellence (NICE) if the ICER is below £20,000 to £30,000 per QALY (McCabe, Claxton, & Culyer, 2008). Probabilistic sensitivity analysis (PSA) was used to estimate parameter uncertainty in the model. This involves multiple, simultaneous draws from probability distributions around uncertain model parameters including transition probabilities, health state values, efficacy and natural recovery (Van Hout, Al, Gilad, Gordon, & Rutten, 1994). The parameter values and distributions and data sources used in the PSA appear in Table 1. Cost-effectiveness acceptability curves (CEAC) are derived from the joint distribution of the difference in costs and differences in effects generated by the PSA. The CEAC shows the probability that CT-PTSD is cost-effective compared to usual care at the NICE threshold and also for a range of alternative willingness to pay thresholds (Fenwick & Byford, 2005). One-way sensitivity analyses were performed testing the inclusion of training costs and a complete case analysis. Trial-based costs and QALYs were adjusted for baseline differences in costs or utility weights, respectively, and potential clinical predictors (age, gender, group) using generalized linear modelling. Missing cost and outcome data were imputed using conditional regression. Analyses were conducted in STATA 14C and Excel.

## Results

### Trial costs

Resource use during the trial and mean costs per child by group are summarized in Table 2. A detailed breakdown of costs at follow up could only be calculated for those children who completed follow-up interviews. Thus, in Table 2 the mean cost of CT-PTSD of £1441 was for the 12 children who were followed up which was slightly lower than the mean cost of the CT-PTSD intervention for all 14 children in the CT-PTSD group which was £1463.

### Trial outcomes

Trial outcomes are presented in Table 3. Cost and QALY data were missing due to noncompletion of questionnaires and losses to follow-up. Missing cost and QALY data were not significantly different between groups (*p* = .366). Accordingly, we imputed missing cost and QALY data using conditional regression.

### Trial based cost-utility analysis

Point estimates for ICERs for complete cases and imputed trial data are presented in Table 4. ICERs in both cases are substantially higher than the recommended threshold of between £20,000 and £30,000 per QALY but interpretation of the ICERs is limited by the short trial time horizon and lack of longer term follow up due to the waitlist control design.

### Model based cost-utility analysis

#### Parameter values

Values for model health states, derived from imputed trial data and baseline data, derived from imputed trial data and baseline data, are presented in Table 5. The initial distribution was 71% PTSD free for treated patients and 27% for untreated patients.

**Figure 3:**
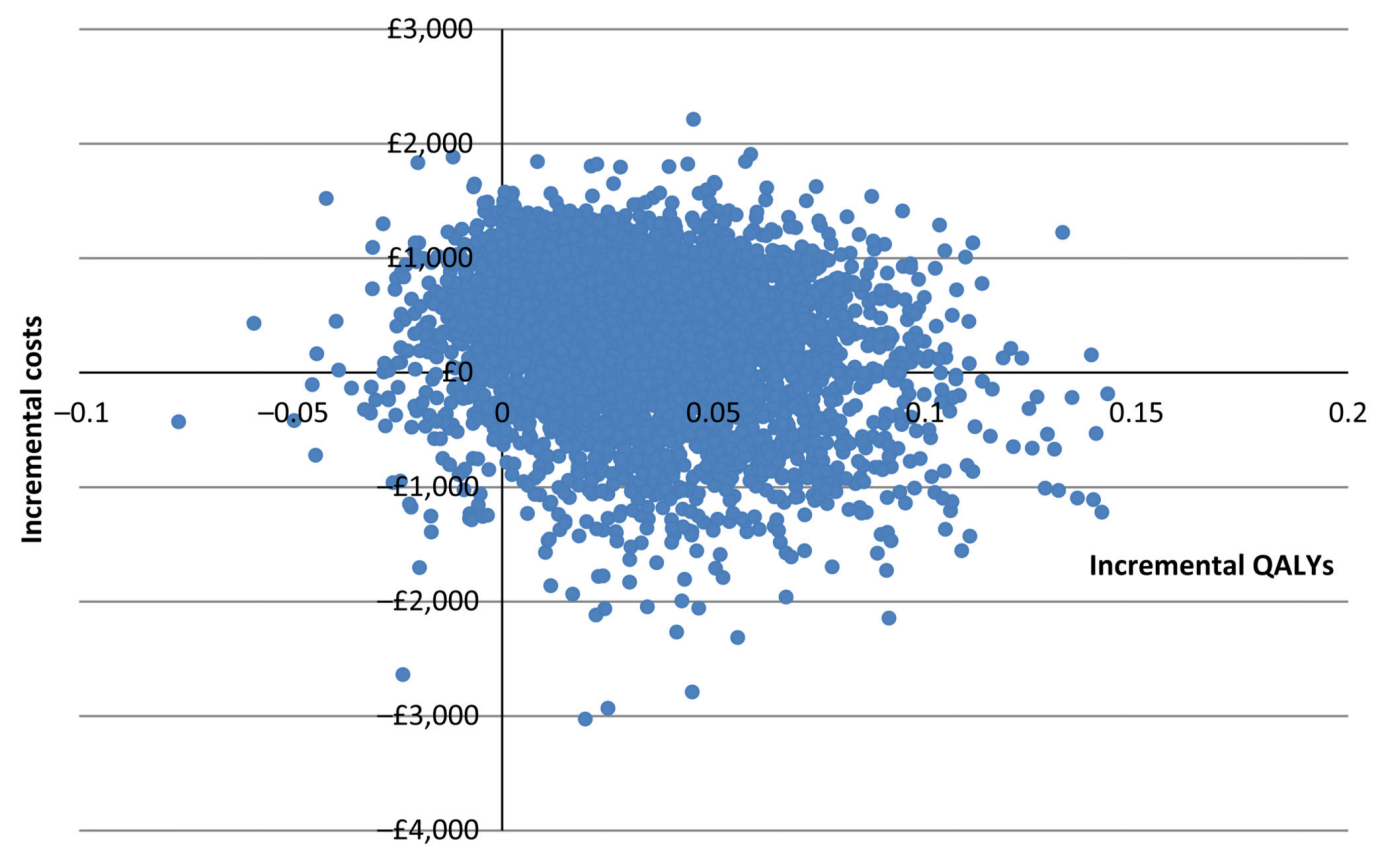
Scatterplot showing the mean differences in total costs and QALYs of CT-PTSD and usual care at 3 years (modelled data) [Colour figure can be viewed at wileyonlinelibrary.com]

**Figure 4:**
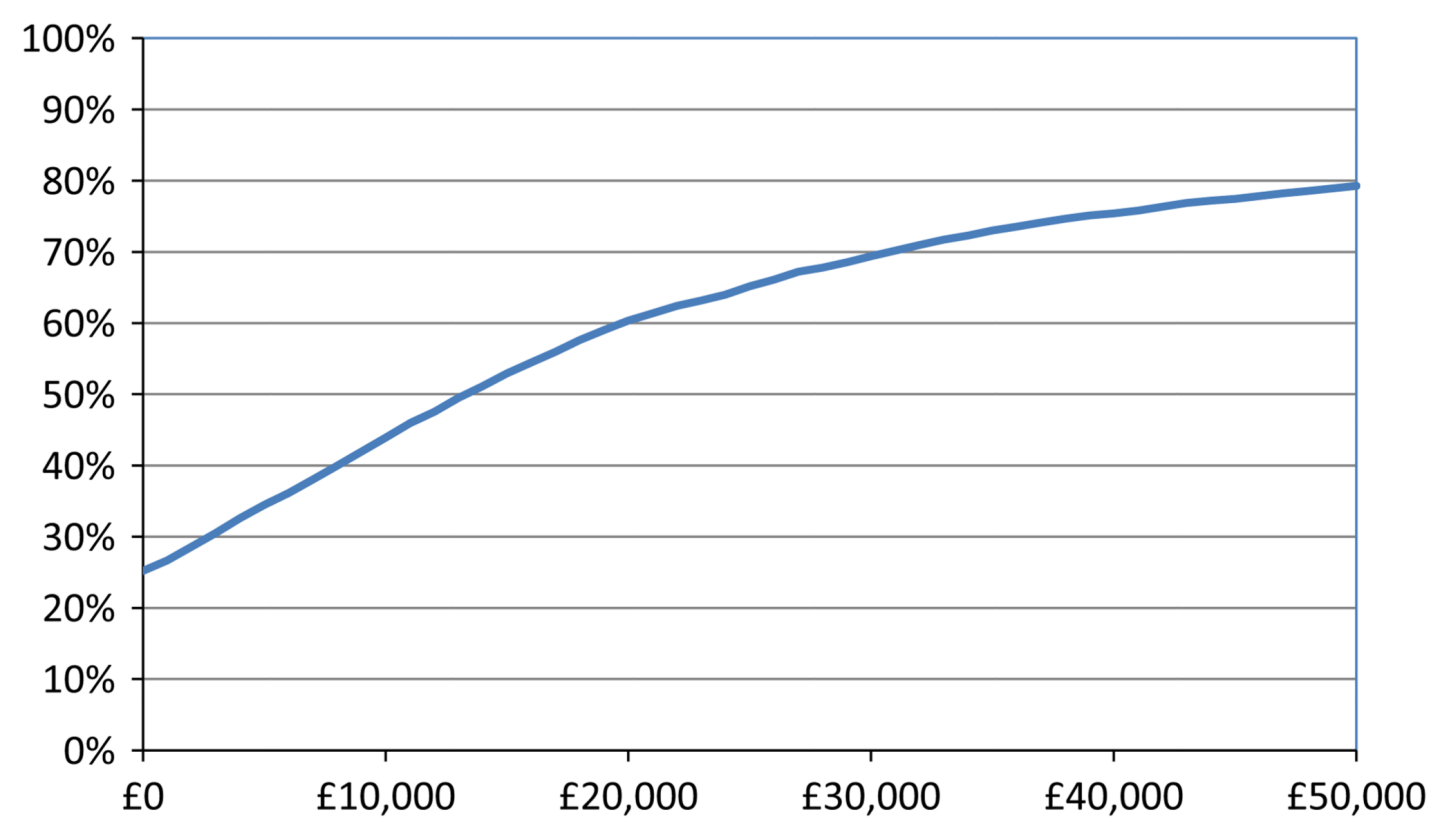
Cost-effectiveness acceptability curve showing the probability (Y-axis) that CT-PTSD is cost-effective compared to usual care for different values (X-axis) a decision maker is willing to pay for an extra QALY [Colour figure can be viewed at wileyonlinelibrary.com]

Point estimates for ICERs based on deterministic, patient-level costs and QALYs at four time points are presented in Table 6. The 3-year ICER was £2,250 per QALY which is well below the NICE threshold of between £20,000 and £30,000 per QALY suggesting that CT-PTSD was cost-effective from the UK NHS perspective compared to usual care. Figure 3 shows 5,000 scatterplots generated by the PSA. Most scatterplots (69%) were in the north-eastern quadrant where the costs and QALYs for CT-PTSD were higher than usual care. Figure 4 is a cost-effectiveness acceptability curve derived from the scatterplot which shows that CT-PTSD has a probability of being cost-effective compared to usual care of 60%–69% at the NICE £20,000 to £30,000 per QALY threshold.

### Sensitivity analysis

#### Complete case analysis

The model based on complete case data differed from the imputed model in terms of the sample size (CT-PTSD = 10, usual care = 11), the initial distribution of recovered patients (CT-PTSD 90%, usual care 18%) and values for costs and QALYs in the PTSD free health state (£264.55, 0.2027 QALYs). The 3-year ICER was £2,806 per QALY which was comparable to the imputed model. PSA showed that CT-PTSD had a probability of being cost-effective compared to usual care of between 69% and 75% at the NICE £20,000 to £30,000 per QALY threshold using only complete case data.

#### Training of therapists

Training costs were excluded from the primary analysis because it was assumed that CT-PTSD specific training would be part of usual professional development. In addition, training and specialist supervision time was only collected for those delivering CT-PTSD; these data were not recorded for the waitlist control group. It may well be, however, that the intervention will require additional training and supervision compared to standard professional development. To reflect benefits for future patients, training costs were amortized over 5 years assuming an annual patient caseload equivalent to the trial recruitment of 29 per annum. This produced an additional cost of £186 per treated patient during the treatment phase. The addition of training costs increased the three year ICER to £16,187 per QALY and reduced the probability of cost-effectiveness compared to usual care to between 51% and 62% at the NICE £20,000 to £30,000 per QALY threshold.

## Discussion

This model-based cost utility analysis, using cost and QALY data collected from a randomized clinical trial extrapolated over the longer term, provides preliminary support that CT-PTSD may be cost-effective from the UK NHS perspective over a three year time horizon. This result was driven by large differences in the proportion of patients who recovered after receiving CT-PTSD (71%) compared to natural remission in the usual care group (27%). Even after factoring continuing natural remission in the first year, the upfront cost of providing CT-PTSD was gradually offset by savings as the proportionally greater number of recovered youths in the treatment group imposed fewer health and social care costs and had better quality of life compared to those with persistent PTSD who were predominantly in the waitlist control condition. The three-year ICER for CT-PTSD compared to usual care control was £2,205 per QALY, which is well below the NICE threshold of £20,000 to £30,000 per QALY. CT-PTSD was likely to be more cost-effective compared to usual care based on probabilistic simulation methods.

In long-term conditions such as PTSD, the main savings and benefits from effective treatments occur well beyond the time horizon of the trial. As a result, trial based cost-effectiveness analyses may underestimate the true cost-effectiveness of new treatments. In this study we extrapolated the trial data using decision analytic methods to model future costs and benefits over a three year period based on the natural history of remission. Other models have modelled economic outcomes over longer periods (5 and 30 years), however, using our trial data, cost-effectiveness became apparent by the third year primarily due to the large initial between-group differences in remission and consequent reductions in healthcare costs associated with untreated PTSD; extrapolation over a longer period would have continued to show improved cost-effectiveness over time.

In this study, children’s health-related quality of life was not directly measured by the CHU-9D utility measure or any other direct measure of utility. The CHU-9D utility values were calculated using a statistical mapping function estimated in an Australian study of 200 caregivers who had completed both the SDQ and the CHU-9D. There are limitations to this approach, including the generalizability of a mapping function based on a sample of Australian parents with children receiving community mental health services to our sample of UK parents with children diagnosed with PTSD. Furthermore, the parent-report SDQ has been found to only have a weak correlation with the symptoms of child-report PTSD (McDermott, Lee, Judd, & Gibbon, 2005) which may underestimate the true impact of symptoms on quality of life and underestimate the cost-effectiveness of CT-PTSD. However, this was the only method available to derive the utility values needed to support this evaluation. We recommend that investigators in future studies include instruments that can directly provide utility values, such as the CHU-9D.

There was uncertainty in the model, particularly around the way that costs and utilities were attributed to the PTSD and PTSD-free health states. In the absence of any UK or comparable international data, we used the baseline results to estimate costs and utilities for children and young people with PTSD persisting 2–6 months after a traumatic event. The utility value of 0.734 was higher than those used in two Australian models which used the literature to estimate lower utility values for adult PTSD-cases (0.57 (Mihalopoulos et al., 2015)) and for untreated sexually abused girls with PTSD (0.61 (Gospodarevskaya & Segal, 2012)) although the Australian estimates included chronic trauma from domestic violence and sexual abuse.

These results come with several important caveats. Most obvious is the small sample size (*n* = 29) and limited follow-up (11 weeks). Recruiting appropriate patients within the narrow treatment window makes research in this population difficult and sample sizes are necessarily small. Economic modelling makes the most of these inherently scarce data. Despite the sample size and follow-up limitations, the treatment effect was significant and treated patients gained more QALYs than untreated ones. The intervention was delivered by clinical researchers and results may be difficult to replicate in general practice. However, when training costs were included in a sensitivity analysis, this did not substantially change the probability (51%–62%) that CT-PTSD was likely to be cost-effective compared to usual care from the NHS/personal social services perspective. Nevertheless, a larger pragmatic trial is needed with longer follow-up, and in more heterogeneous populations such as urban areas, to confirm effectiveness, generalizability and cost-effectiveness.

## Figures and Tables

**Table 1:** Parameter values, sources and uncertainty distributions for the PSA

Parameter	Values (95% CI)	Distribution	Source
CT-PTSD efficacy	71% (58%–92%)	Beta(α 19 β 7)	Trial data
Usual care efficacy	27% (8%–55%)	Beta (α 3 β 9)	Trial data
Remission (between 3 months and year 1)	34% (21%–49%)	Beta (α 14 β 95)	Hiller et al. (2016)
Remission (years 2 & 3)	0%	–	Hiller et al. (2016)
PTSD health state costs	£549 (£377–£721)	Gamma(α 19.532 γ 28.118	Trial data
PTSD free costs	£236 (£93–£379)	Gamma (α 10.369 γ 22.738)	Trial data
PTSD heath state QALY	0.185 (0.158–0.212)	Beta (α 2618 β 10940)	Trial data
PTSD free QALYs	0.193 (0.186–0.199)	Beta (α 808 β 3567)	Trial data

PSA, Probabilistic sensitivity analysis; PTSD, post-traumatic stress disorder; QALYs, quality-adjusted life years.

**Table 2:** Disaggregated, unadjusted mean (standard deviation) costs by group and time (complete cases) for the trial period

	Baseline		Follow-up	
	CT-PTSD (*n* = 14) Mean £ (*SD*)	Usual care (*n* = 15) Mean £ (*SD*)	CT-PTSD (*n* = 12) Mean £ (*SD*)	Usual care (*n* = 11) Mean £ (*SD*)
CT-PTSD	0 (0)	0 (0)	1441 (809)	0 (0)
Inpatient	0 (0)	0 (0)	0 (0)	0 (0)
Outpatient	68 (176)	82 (172)	48 (104)	86 (238)
Emergency Department	37 (71)	17 (35)	0 (0)	0 (0)
Ambulance	100 (191)	47 (93)	0 (0)	42 (139)
Total hospital services	205 (355)	146 (188)	48 (104)	128 (260)
GP home visit	78 (128)	98 (247)	30 (101)	0 (0)
GP surgery	110 (182)	89 (114)	45 (50)	104 (119)
GP Phone	0 (0)	0 (0)	9 (24)	10 (32)
GP Nurse	1 (2)	4 (10)	1 (3)	0 (0)
District nurse	17 (51)	4 (16)	36 (100)	13 (28)
Paediatrician	0 (0)	3 (12)	0 (0)	4 (14)
Clinical psychologist	0 (0)	23 (54)	6 (19)	0 (0)
CAMHS worker	0 (0)	31 (66)	0 (0)	42 (132)
Counsellor	43 (86)	44 (108)	17 (31)	19 (57)
Educational psychologist	30 (107)	27 (100)	0 (0)	0 (0)
Advice service	2 (6)	44 (148)	0 (0)	9 (22)
Social services	0 (0)	0 (0)	25 (16)	19 (58)
Other services	32 (110)	72 (468)	14 (45)	0 (0)
Medications	19 (71)	0 (0)	19 (63)	4 (11)
Total community services	332 (83)	432 (119)	202 (47)	223 (59)
Total costs	537 (102)	578 (119)	1691 (532)	351 (392)

GP, general practitioner; CAMHS, child and adolescent mental health service; PTSD, post-traumatic stress disorder.

**Table 3:** Complete case and imputed trial outcomes by group

Outcome	CT-PTSD	Usual care	Unadjusted difference
Complete case	*n* = 10	*n* = 11	
PTSD cases; *n* (%)	1 (10%)	9 (82%)	−72%
QALYs; mean (*SD*)	0.1933 (0.0119)	0.1846 (0.0196)	.0087
Imputed data	*n* = 14	*n* = 15	
PTSD cases; *n* (%)	4 (29%)	11 (73%)	−44%
QALYs; mean (*SD*)	0.1979 (0.0137)	0.1823 (0.0188)	.0156

PTSD, post-traumatic stress disorder; QALYs, quality-adjusted life years.

**Table 4:** Trial based cost utility analysis

	Costs CT-PTSD Mean £ (*SD*)	Usual care Mean £ (*SD*)	Adjusted difference	QALYs CT-PTSD Mean (*SD*)	Usual care Mean (*SD*)	Adjusted difference	ICER £ per QALY
Complete case	£1,691 (£532)	£351 (£392)	£1,284	0.1929 (0.0108)	0.1851 (0.0201)	.0103	£124,660
Imputed	£1,686 (£549)	£307 (£352)	£1,346	0.1979 (0.0137)	0.1823 (0.0186)	.0095	£141,684

ICER, incremental cost- effectiveness ratio; PTSD, post-traumatic stress disorder; QALYs, quality-adjusted life years.

**Table 5:** Estimated annual health state values

Health state	Costs	QALYs
PTSD free	£1,114	.7725
PTSD	£2,596	.7386

PTSD, post-traumatic stress disorder; QALYs, quality-adjusted life years.

**Table 6:** Model based cost-utility analysis

	Costs			QALYs				
	CT-PTSD	Usual care	Difference	CT-PTSD	Usual care	Difference	ICER (£ per QALY)	CEAC (*p*)^a^
Trial	£1,686	£307	£1,346	0.198	0.182	.0095	£141,684
Year 1^b^	£2,598	£1,540	£1,058	0.773	0.748	.0246	£42,967	4%–19%
Year 2	£3,752	£3,125	£627	1.557	1.522	.0352	£17,779	31%–45%
Year 3	£4,865	£4,768	£97	2.370	2.324	.0577	£2,205	60%–69%

a Probability that CT-PTSD is cost-effective at the NICE threshold of £20,000 to 30,000 per QALY.

b Year 1 data consist of 3-month trial data and 9-month modelled data. ICER, incremental cost- effectiveness ratio; PTSD, post-traumatic stress disorder; QALYs, quality-adjusted life years.

## Key Points

- Untreated PTSD in children and adolescents is a burden on health systems and families.
- Early intervention with Cognitive Therapy has been found to be efficacious in helping children and adolescents to recover from PTSD.
- The long-term cost-effectiveness of interventions for PTSD in children and adolescents is important from the policy and practice perspectives.
- CT-PTSD is potentially a cost-effective use of scarce NHS resources.
